# Urinary Malondialdehyde (MDA) Concentrations in the General Population—A Systematic Literature Review and Meta-Analysis

**DOI:** 10.3390/toxics10040160

**Published:** 2022-03-29

**Authors:** Antonio Toto, Pascal Wild, Mélanie Graille, Veronica Turcu, Camille Crézé, Maud Hemmendinger, Jean-Jacques Sauvain, Enrico Bergamaschi, Irina Guseva Canu, Nancy B. Hopf

**Affiliations:** 1 Center for Primary Care and Public Health (Unisanté), University of Lausanne, Route de la Corniche 2, 1066 Lausanne, Switzerland; antonio.toto@unisante.ch (A.T.); pascal.wild@unisante.ch (P.W.); melanie.graille@unisante.ch (M.G.); veronica.turcu@unisante.ch (V.T.); crezecamille@gmail.com (C.C.); maud.hemmendinger@unisante.ch (M.H.); jean-jacques.sauvain@unisante.ch (J.-J.S.); irina.guseva-canu@unisante.ch (I.G.C.); 2 Laboratory of Toxicology and Industrial Epidemiology, Department of Public Health and Pediatrics, University of Turin, Via Zuretti 29, 10125 Turin, Italy; enrico.bergamaschi@unito.it

**Keywords:** oxidative stress, MDA, systematic review, meta-analysis, urinary biomarker, reference range, general population

## Abstract

Oxidative stress has been associated with various inflammation-related human diseases. It is defined as an imbalance between the production and elimination of reactive oxygen species (ROS). ROS can oxidize proteins, lipids, and DNA, and some of these oxidized products are excreted in urine, such as malondialdehyde (MDA), which is considered a biomarker for oxidative damage of lipids. To interpret changes of this biomarker as a measure of oxidative species overproduction in humans, a background range for urinary MDA concentration in the general population is needed. We sought to establish urinary MDA concentration ranges for healthy adult populations based on reported values in the available scientific literature. We conducted a systematic review and meta-analysis using the standardized protocol registered in PROSPERO (CRD42020146623). EMBASE, PubMed, Web of Science, and Cochrane library databases were searched from journal inception up to October 2020. We included 35 studies (divided into 47 subgroups for the quantitative analysis). Only studies that measured creatinine-corrected urinary MDA with high-performance liquid chromatography (HPLC) with mass spectrometry (MS), fluorescence detection, or UV photometry were included. The geometric mean (GM) of urinary MDA concentration was 0.10 mg/g creatinine and 95% percentile confidence interval (CI) 0.07–0.12. Age, geographical location but not sex, and smoking status had a significant effect on urinary MDA concentrations. There was a significant increasing trend of urinary MDA concentrations with age. These urinary MDA values should be considered preliminary, as they are based on mostly moderate to some low-quality evidence studies. Although urinary MDA can reliably reflect excessive oxidative stress in a population, the influence of physiological parameters that affect its meaning needs to be addressed as well as harmonizing the chemical analytical methods.

## 1. Introduction

**Figure 1:**
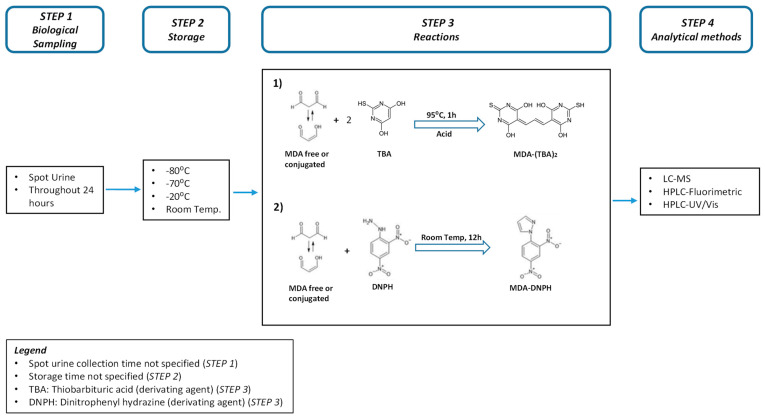
Simplified schematic process for the determination of MDA in biological samples (Urine).

Oxidative stress is defined as an imbalance between the production and elimination of reactive oxygen species (ROS) in the body [1]. ROS targets many biological entities but mainly lipids and polyunsaturated fatty acids [2]. When these biomolecules undergo peroxidation, the chain reaction evolves in three steps: initiation, propagation, and termination, with various reactive products generated at each step [3]. Malondialdehyde (MDA) (chemical structure shown in Figure 1), together with other aldehydes, is one of polyunsaturated fatty acids’ peroxidation best-studied end-products [4].

MDA can be generated both through an enzymatic pathway, identical to thromboxane A2 and prostaglandins, as well as through a non-enzymatic process [5]. MDA is not just a biomarker of oxidative stress but also a biologically active compound having several biological roles [5,6]. Owing to its several biological functions, MDA can also be regarded as a biomarker of peroxidation of cell membrane fatty acids when produced via the enzymatic process [6]. MDA can act as a signaling messenger in insulin secretion [7] and as an inducer of collagen-gene expression in hepatic cells [8]. MDA generated via a non-enzymatic process would, however, interact with other biomolecules, such as proteins, amino groups, and DNA [9], to generate a multitude of adducts, ultimately resulting in a genotoxic effect. MDA has been indicated as putatively being the most mutagenic molecule among ROS end-products [4].

Circulating MDA can be detectable either free (unconjugated) or conjugated [10], and the sum of the two forms is labeled total MDA. MDA forms adducts with many biological molecules, and the majority of MDA produced is found in the conjugated form. MDA adducts are highly immunogenic, i.e., able to trigger an immune response. They have been found to be associated with autoimmune diseases, such as lupus erythematosus and nephritis [11], while others have shown a correlation with the development and progression of atherosclerosis [12] and longevity [5].

MDA concentrations in different biological samples collected from several sub-populations have been investigated. Plasma MDA levels tend to be higher in smokers than in non-smokers and in populations exposed to high compared to low air pollution [13]. Plasma MDA levels are consistently higher in patients with acute stroke [14], diabetes, and chronic inflammation [10], such as chronic obstructive disease (COPD) [15] and asthma [13] compared with healthy people. Urinary MDA levels are mostly evaluated as a biomarker of systemic oxidative stress and found to be elevated in conditions such as urinary infections [16], diabetic nephropathy [17], but also after air pollution exposure [18]. Body mass index (BMI), sex, and age have been mentioned as potential confounding factors in a sizable part of the literature.

Urinary MDA concentrations are quantified either by immunochemical assays or chemical analytical methods. Figure 1 outlines the different steps generally needed for urinary MDA quantification, i.e., urine sample collection (step 1), storage and transportation (step 2), and derivatization of urinary MDA with a complexing agent (step 3). This step is needed to increase sensitivity and reduce the limit of detection. Both thiobarbituric acid (TBA) and dinitrophenyl hydrazine (DNPH) are used as derivatization reagents. TBA is less specific compared to DNPH [19]. The last step is the quantification of MDA with a chemical analytical method (step 4) [6,20].

Collecting urine samples is easy, convenient, and non-invasive. These are reasons why urinary MDA is an interesting quantifiable biomarker of systemic oxidative stress. To use this biomarker tool to measure oxidative stress in selected populations, we need a general population reference range. As of yet, a general population reference range has not been generated. In addition, urinary MDA concentration ranges reported for the general population in the scientific literature vary greatly and are inconsistent [21]. We, therefore, sought to close this data gap by performing a systematic review and meta-analysis [22] according to our registered Prospero protocol ((https://www.ncbi.nlm.nih.gov/pmc/articles/PMC7311981/pdf/ijms-21-03822.pdf) Section 4.1), and providing reference ranges for urinary MDA concentrations in a healthy adult population. Our aim was two-fold:

*Aim 1: Provide the geometric mean (GM) and standard deviation (GSD) for urinary MDA concentrations,*

*Aim 2: Assess the influence of age, smoking status, geographic locations (countries), and sex on urinary MDA concentrations.*

## 2. Materials and Methods

We registered our study protocol in the International Prospective Register of Systematic Reviews (PROSPERO; registration number CRD42020146623). We report our results here following the recommendations from the Preferred Reporting Items for Systematic Reviews and Meta-Analysis (PRISMA) [23].

### 2.1. Literature Search

We searched the published scientific literature from journal inception and up to October 2020 in the following bibliographic electronic databases: EMBASE, PubMed, Web of Science, and Cochrane library. The full search strategy, including the search string used, can be found in (https://www.doi.org/10.16909/dataset/17, accessed on 20 February 2022). Only original research studies written in either English or French were included. Two researchers (CC and AT) conducted two rounds of selection, i.e., abstract screening and full-text reading. We excluded studies without quantitative data for MDA, non-human studies, reviews, correspondence, conference papers, expert opinions, and editorials, as well as abstracts without full text. The reviewers (CC and AT) independently performed a first screening of titles and abstracts retrieved during the searches, using Rayyan software [24], a systematic review web application for title and abstract screening [25].

### 2.2. Study Selection

**Figure 2:**
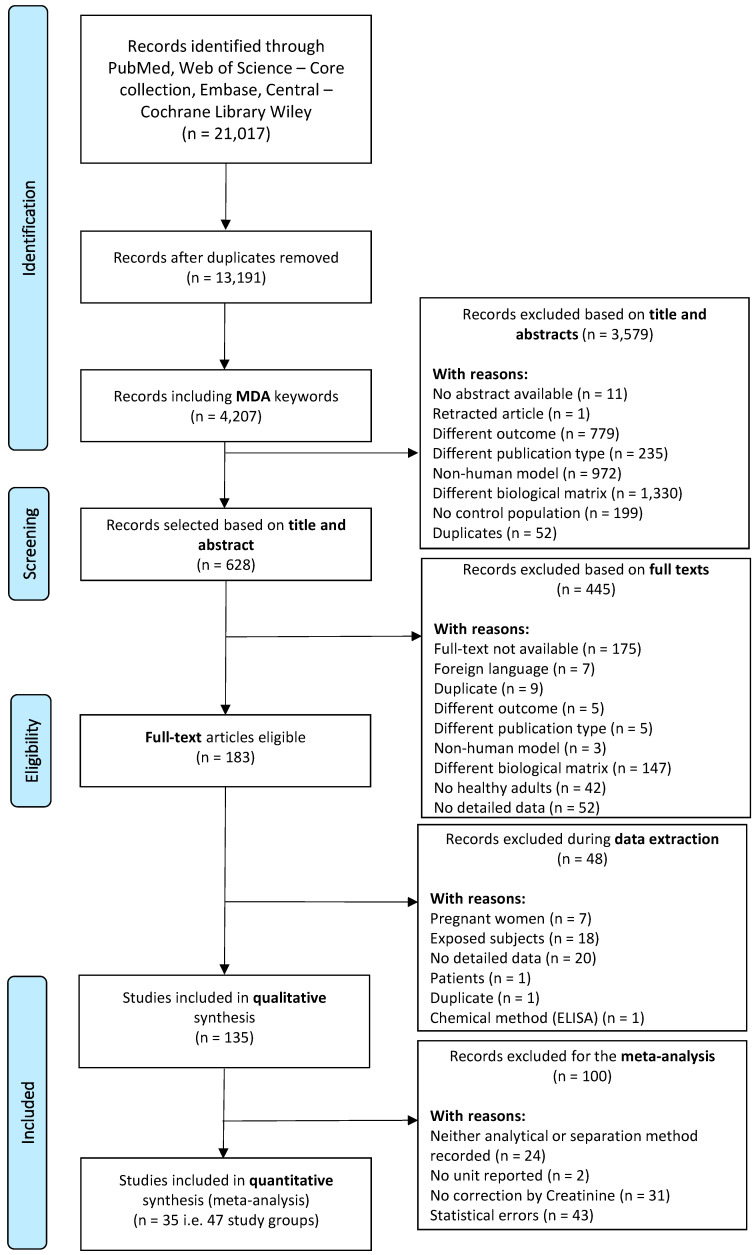
Flow chart describing the selection process of the 135 studies included in the quality assessment and the subset of 35 studies included in this meta-analysis.

We only included original research studies conducted on healthy adult human participants (aged > 18 years, no known disease), measuring urinary MDA. The flow chart outlining the study selection is presented in Figure 2. An initial 21,017 records were retrieved and exported to the Rayyan software. We excluded 3579 articles after abstract screening. Studies showing non-creatinine-adjusted data and values with suspected unit mistakes were excluded during the second round of selection. Overall, 135 studies were deemed eligible, and the corresponding papers were downloaded into the EndNote software. We conducted a standardized quality assessment, which was developed as part of our study protocol [26]. In a third selection round, we selected studies purposely for the quantitative (meta-)analysis. We thus excluded studies reporting no standard deviations (SD) or confidence intervals (CI) with their mean and median values, suspiciously low coefficient of variation (CV < 20%), missing chemical analytical method entirely or descriptions partially missing, such as separation techniques and detection method, and aberrant units. A total of 35 articles remained comprised of 47 different exposure groups, which we carried forward to the meta-analysis.

Figure 2 flow chart describing the selection process of the 135 studies included in the quality assessment and the subset of 35 studies included in this meta-analysis.

### 2.3. Data Extraction

We used the standardized data extraction form developed as part of our study protocol [26]. In addition to populations with known diseases, groups with known occupational and/or environmental chemical exposures were also excluded. When data on several subgroups were available in the published article, we extracted all subgroup-specific data. Only baseline data were extracted whenever data from several time points were available. We also recorded possible covariates that affect the oxidative stress concentrations such as study design, sample collection methods (spot urine samples or 24 h urine samples), sample storage, pre-analytical methods, and vitamin supplements, as well as statistical analysis. A statistician (PW) cross-checked all data extracted for the meta-analysis.

### 2.4. Quality Assessment

Whether a qualitative or a quantitative approach is most appropriate depends on the nature and state of the existing literature, the research questions, and theoretical and empirical issues. We used a standardized quality assessment checklist previously used in other studies [26,27]. Briefly, the quality checklist covered four domains: (I) study sample, (II) study design and risk of bias, (III) technical and analytical methods, (IV) data processing, analysis, and result reporting. We assessed each domain based on a number of objective criteria (Supplementary Table S1) by grading these criteria with sub-scores from 1 to 3. The resulting sub-scores were first summarized in a quality score for each of the four domains, then into an overall study quality score as described in the GRADE guidelines [28]. The total quality scores ranged between 9 and 27 (Supplementary Table S2) and were considered “high” when scores were equal or higher than 20, “moderate” between 14 and 19, and “low” for equal or lower than 13 [28]. The quality assessments of the included studies were performed by one (AT) and reviewed by two independent reviewers (NBH, IGC).

### 2.5. Statistical Analysis

Values of urinary biomarkers are generally log-normally distributed; we, therefore, computed geometric means (GM) and geometric standard deviations (GSD) as the basis for the meta-analysis, i.e., equivalently *mu*L (log geometric means (GM)) = ln(GM) and *sdL* (log geometric standard deviations (GSD)) = ln(GSD). Details of the computations are given in Graille et al. 2020 [27]. All study-specific results were then converted to mg/g creatinine using the molar weights of MDA and creatinine when necessary.

The chemical analytical methods used were gas chromatography (GC) and high-performance liquid chromatography (HPLC) separation with either mass spectrometry (MS), fluorescence, and spectrophotometry (UV) detection (we did not include immunohistochemistry analyses, e.g., ELISA, but focused on chemical analytical quantification). Forest plots were used to display GMs and 95% confidence intervals (in mg/g creatinine) of the different study groups both graphically (the squares represent the GM and the lines around the CI) with the study groups re-grouped by age and smoking categories, respectively. The diamonds represent the summary GM of the categories. Between-study heterogeneity was assessed using the Q test for homogeneity within each category and displayed on the forest plots [29]. If the between-study heterogeneity is larger than the between-subject heterogeneity, then any attempt of obtaining a summary value for individual participants will not be valid. We further modeled the study group-specific log-transformed GMs using a linear mixed model with the study ID as a random effect, without further considering the within-study heterogeneity. Such an analysis is warranted when the between-study heterogeneity dominates the within-study heterogeneity. The study ID as a random effect was included in order to account for between-study heterogeneity when assessing the effect of other parameters. We used STATA, version 16 software for data management and statistical analysis.

## 3. Results

**Figure 3:**
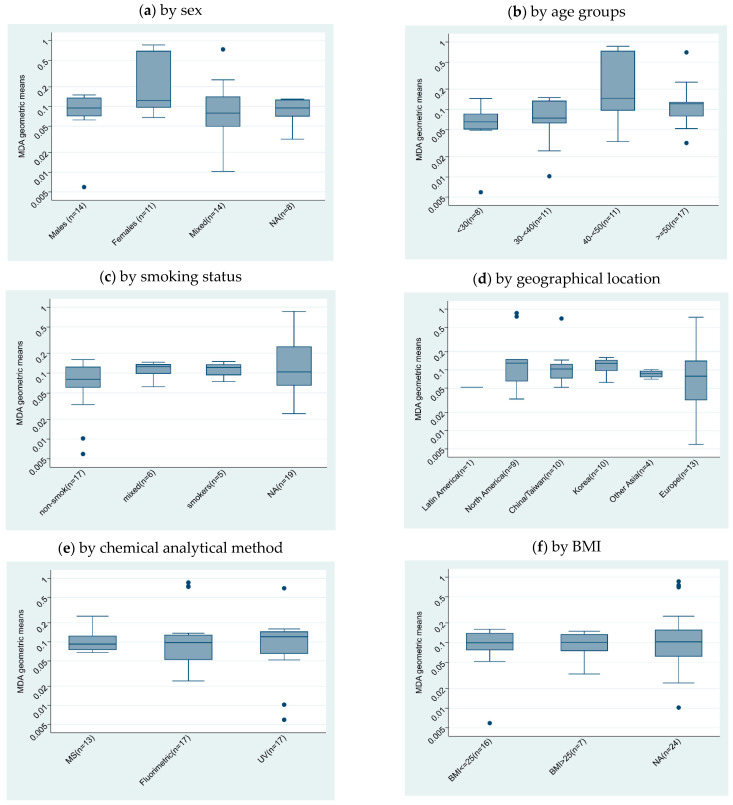
Boxplots of log-transformed urinary MDA concentration (mg/g creatinine) GM (*y*-axis) by subgroups (*x*-axis): (**a**) sex, (**b**) age, (**c**) smoking status, (**d**) geographical location, (**e**) chemical analytical method, and (**f**) BMI.

We included 135 studies in the qualitative analysis, with a subset of 35 studies in the quantitative analysis (Figure 2). We described the subgroups by sex (Figure 3a), age (Figure 3b), smoking status (Figure 3c), geographical location (Figure 3d), chemical analytical methods (Figure 3e), and body mass index (BMI) (Figure 3f) subgroups, as far as these results were available, which resulted in the description of 47 study subgroups.

We combined the chemical separation methods (LC and GC) as they did not show significantly different results (Figure 3e). We merged UV/VIS and photometry detection and labeled this “UV/VIS”. Consequently, the chemical analytical methods are represented with their detection method in Figure 3e. We were unable to include chemical analytical variables such as derivatization method, clean-up procedures, or instrumental parameters in our analysis as these were not reported in most studies.

The chemical analytical methods were comparable (Figure 3e); thus, we did not include this variable in the statistical analysis. This was also true for BMI (Figure 3f); thus, we did not include this variable either. Smoking status, age group, and geographical location were variables included in the statistical models.

The overall GM and 95% CI for urinary MDA are provided in Table 1, as well as the GM and 95% CI for each age group. The overall between-study 95% reference range of the study-specific GMs 0.01–0.65 (data not shown) is, of course, much wider.

Table 2 gives the results from the mixed-effect regression analysis. The majority of the studies were conducted in Europe (*n* = 23) with North America, China/Taiwan, and Korea with half as many studies. Urinary MDA concentrations were seldom reported for Africa and Latin America. Geographical location has an impact on healthy populations in Asian countries (China, Korea, and Taiwan), having higher urinary MDA levels than in the European studies. The test for trend with age group with higher urinary MDA concentrations in older participants was significant (*p* = 0.041). Smoking status had a significant effect, even though this effect was due to differences with the included studies that did not stratify participants by smoking status or report smoking status. Since age and smoking status were the most relevant factors as seen from the statistical analysis, we present the data as forest plots according to these two factors.

**Figure 4:**
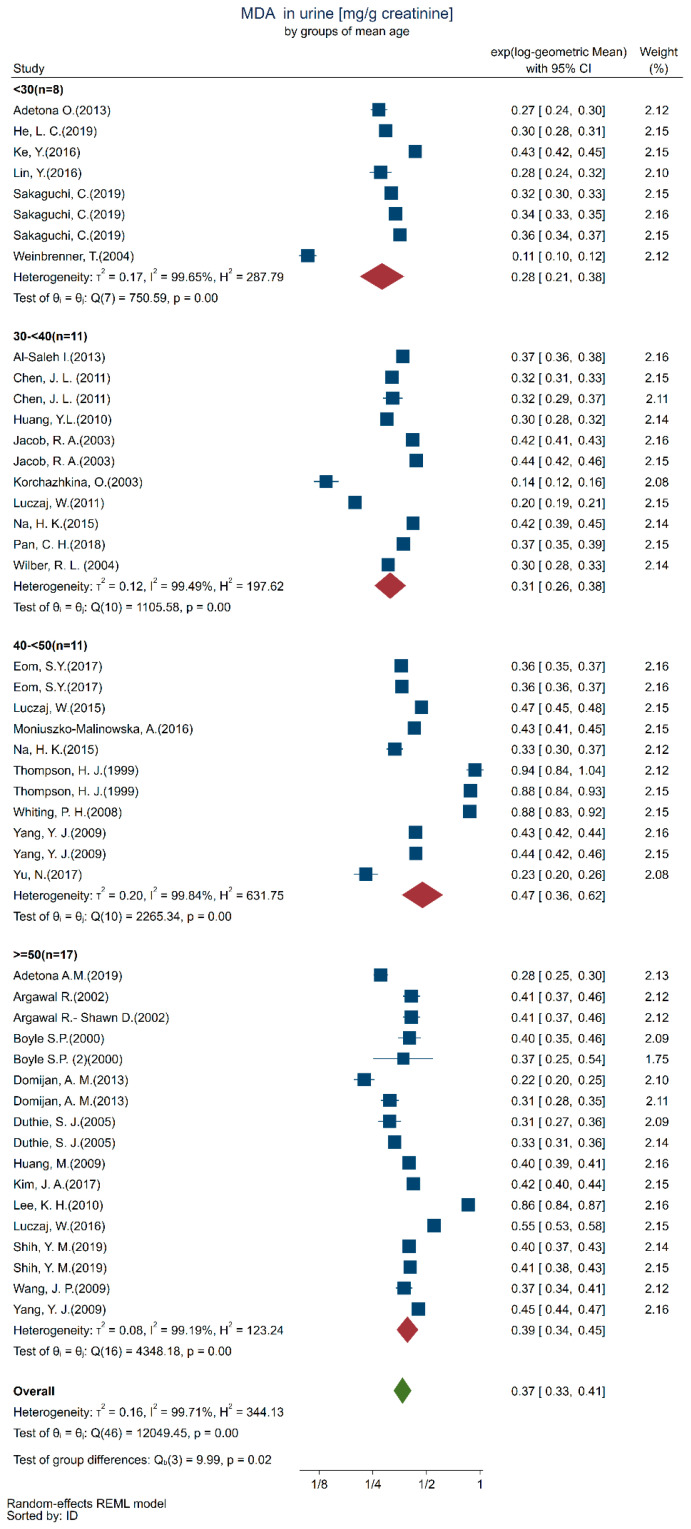
Forest plot of urinary MDA concentrations quantified in healthy adults (18+ years) participants by age groups (<30: [30,31,32,33,34,35], 30–<40: [36,37,38,39,40,41,42,43,44], 40–<50: [42,45,46,47,48,49,50,51], >=50: [7,46,50,52,53,54,55,56,57,58,59,60,61,62]).

**Figure 5:**
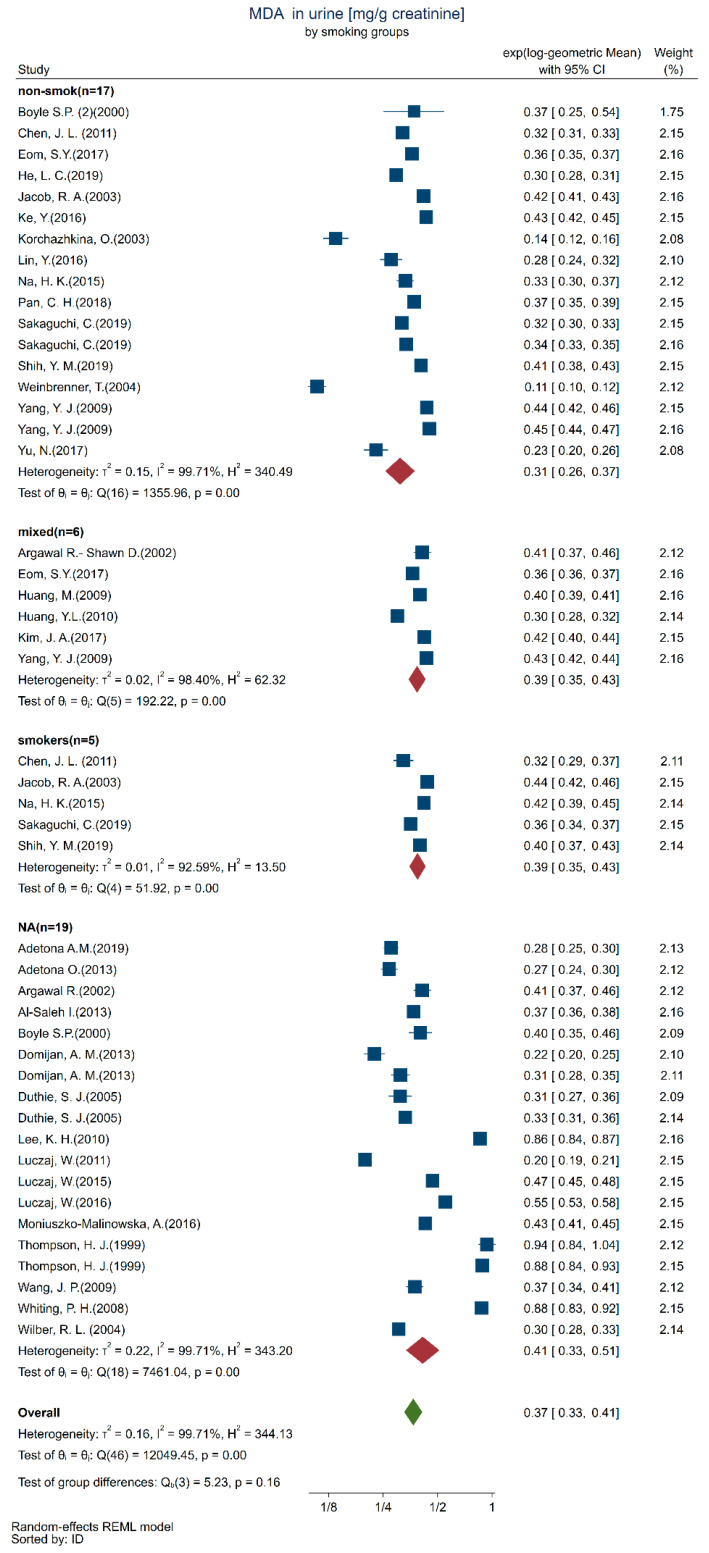
Forest plot of urinary MDA concentration (mg/g creatinine) in healthy adult (18+ years) by smoking/non-smoking groups (non-smok: [31,32,33,34,35,37,39,40,42,43,45,50,51,56,62], mixed: [38,45,50,54,59,60], smokers: [34,37,39,42,62], NA: [7,30,36,41,44,46,47,48,49,52,53,55,57,58,61,63].

We present the results in separate forest plots of urinary MDA concentrations (mg/g creatinine) in Figure 4 by age groups and Figure 5 by smoking/non-smoking groups. It is apparent from these forest plots that the between-study groups heterogeneity was much larger than the within-study heterogeneity uncertainty to the point that the within-study confidence intervals are barely distinguishable. This is confirmed by the highly significant Q tests within each all the smoking and age categories.

## 4. Discussion

### 4.1. Interpretation of Findings

From the analysis of the literature, we can extrapolate and suggest a mean value of 0.10 mg/g creatinine and an overall reference range of 0.01–0.65 mg/g creatinine for study-population GMs of free urinary MDA concentrations in healthy adults. These values are valid for chemical analytical methods and not necessarily for colorimetric assays (e.g., ELISA).

The urinary MDA concentrations increase with age, which corroborates previous findings [64]. We were able to analyze the age factor in our meta-analysis as most of the included studies (77%) had recorded the age of their subjects. This age-related trend for increasing urinary MDA concentrations might reflect an increase in oxidative stress, which is expected with aging. This has previously been thoroughly discussed [65] in a joint effort from several researchers studying oxidative stress and health-related outcomes as well as the underlying biochemical mechanisms. Aging has been found to be related to the dysfunction of proteasome-mediated degradation of oxidized proteins, a critical player for protein homeostasis maintenance. Yet, proteasome up-regulation has been shown to successfully decelerate the aging progression by enhancing resistance to oxidative stress in genetically modified animals. Thus, increases in MDA levels with age might reflect increased oxidative stress through the progressive dysfunction of the protective proteasome pathway [66].

### 4.2. Heterogeneity

We found an overall high heterogeneity in studies included in this systematic review. A number of unknown factors, e.g., vitamin supplements, biological variability, air pollution, also contribute to the modification of oxidative stress levels that have yet to be characterized. These unknowns probably contributed to the great variability in our meta-analysis [67,68]. The lack of information on these covariates led to quality scores in moderate (65% of the studies were scored as moderate) and low (35%) levels. The heterogeneity could be related to study designs, sample collection methods (spot urine samples or 24 h urine samples), sample storage, pre-analytical methods, and statistical analysis. We included only 26% (35 out of 135) of the selected studies in the quantitative synthesis. Indeed, we discarded 32% due to statistical errors; i.e., presence of extreme values (outliers); or undescribed data distribution (GSD, IQR, CI were missing, coefficient of variations either too high >300% or too low <20%), 23% of the studies did not have their data creatinine-corrected, 1% had no units, and 18% did not have either a complete chemical analytical or separation method description.

#### 4.2.1. Heterogeneity in Data Collection of Demographics

We found greater urinary MDA concentrations in the Asian populations. In spite of no apparent reasons, environmental factors and dietary habits may account for differences between Asian and Western countries. Air pollution measured as particular matter with an aerodynamic diameter of 2.5 μm or smaller (PM2.5) has been associated with greater urinary MDA concentrations [69], but so has other geographically linked parameters such as vitamin intake [67,68]. It is therefore difficult at this stage to assess what role geographical location plays with regard to other factors.

An increased urinary MDA value with increasing BMI has been reported [70]. However, we could not assess this effect in our meta-analysis, as BMI was missing in 54% of the included studies. Sex differences in MDA concentrations have been demonstrated in healthy adults [71]. For instance, urinary MDA concentrations were reported to be higher in healthy young men compared to age-matched women [72]. Three-quarters of the studies reported separate values for men and women; however, we could not detect a sex difference in urinary MDA levels.

Smoking is considered a source of oxidants leading to lipid peroxidation and a factor depleting antioxidants. Even though urinary and plasma MDA concentrations are associated, and plasma MDA concentrations have been shown to be different in smokers and non-smokers [73,74], we did not find a clear difference in smoking status, albeit the median urinary MDA concentration values seemed slightly higher among smokers. Other studies [34,75] have shown urinary MDA concentrations to be significantly greater in smokers compared to non-smokers. The lack of observed difference between these groups in our review might be related to the number of cigarettes smoked, as suggested in a previous review [74], which found a dose-dependent relationship between cigarette smoke exposure and plasma MDA concentration. Furthermore, urinary MDA might not be a sensitive biomarker for detecting the increase in oxidative stress from tobacco smoking [76], as studies show divergent results. This has been suggested by other authors [77]. We believe one reason might be that the use of thiobarbituric acid (TBA) as a derivatization agent in the analysis is not sufficiently sensitive. Although no difference could be detected between the groups of smokers, the groups of non-smokers, and the mixed smokers/nonsmoker groups in our review, the MDA concentration was higher in nearly one-half of the study groups for which the smoking status was not reported, leading to overall statistical significance. Consequently, we cannot rule out that smoking has an effect on urinary MDA levels.

#### 4.2.2. Heterogeneity in Collection of Biological Samples

Most of the studies used spot urine samples (69 study groups; some of them without any indication of collection time) rather than 24 h urine collection (4 study groups). MDA levels fluctuate [78] during the day depending on activity, and urine concentrations represent MDA excretions from the last urine void until the next. The first urine void provides a measure of cumulative MDA concentrations (representing excretion overnight) and correlates well with 24 h urinary collections [27]. We included studies reporting any spot or 24 h urine samples. This difference in urine collection time probably contributed to the heterogeneity of the results. We found varying storage temperatures, and information on storage time and conditions were often omitted (56 creatinine-corrected studies). Storage conditions might have an effect on the measured concentration of MDA, knowing that urinary MDA concentrations need to be analyzed within 24 h of collection and stored in an airtight container at 0 °C [40]. In fact, one study [40] has shown a 43% ± 15% reduction in MDA concentration when the urine sample was left at −20 °C for more than 3 weeks. This decay needs to be confirmed, and standardized storage methods need to be developed, as most population studies cannot analyze the urine sample immediately after collection.

#### 4.2.3. Heterogeneity in MDA Analysis

The included studies reported using different derivation methods for MDA analysis, which probably contributes to the overall heterogeneity. The most common, e.g., 96% of the included studies used the TBARS derivatization method, which quantifies TBARS formed as a byproduct of lipid peroxidation. This method of assay requires high temperatures (80–100 °C) for an extended incubation time under strong acidic conditions. These harsh conditions can lead to reactions with several other materials such as non-lipid-related materials and fatty peroxide-derived decomposition products. Consequently, urinary MDA is overestimated using TBA by a factor of almost 10 compared to another derivatization agent 2,4 dinitrophenylhyldrazine (DNPH) [40]. DNPH requires lower temperatures (37 °C) and slightly acidic pH, which should lead to results that are more specific. The DNPH derivatization method was used only in 4% of cases of all selected studies. Therefore, we could not compare the results from different derivatization methods. We recommend that future studies use specific derivatizing agents such as DNPH.

We found that authors reported urinary MDA concentrations either as free, conjugated or total, but this was rarely specified in the studies. This can contribute to the observed heterogeneity as well. If this specification was reported, then authors more often reported free MDA over total MDA as a biomarker of oxidative stress. Total urinary MDA would better estimate the total body burden, but the ease and the convenience of only quantifying free MDA, skipping the hydrolysis step will not just shorten the analysis time but also the cost. Urinary concentrations of free MDA and total MDA are reported to be significantly correlated [13]; thus, the use of free or total MDA may reflect similar oxidative stress levels.

One limitation of our study is that we did not systematically record results from studies using ELISA. Chemical analytical methods and colorimetric assays, and ELISA have not been compared for urinary MDA analysis. Analytical methods have been shown to provide different values for other oxidative stress biomarkers such as 8-isoprostane [27]. We cannot compare urinary MDA concentrations between ELISA and chemical analytical methods, as we did not include ELISA in our meta-analysis.

Another limitation is that we were unable to read articles that were not in English or French. We, therefore, do not know how many articles with relevant information we missed.

### 4.3. Recommendations

We believe that the between-studies heterogeneity can be reduced and controlled if future studies will address the effects of additional factors of interest, such as biological mechanisms and physiological variables. Studies should clearly state whether they have quantified total MDA or free MDA in urine. Harmonizing the unit metrics (mg/g creatinine) for reporting urinary MDA would also be helpful. The variation in urine flow rate, body mass, and workload could certainly affect urinary MDA values, as is common for other effect biomarkers. For this purpose, urinary creatinine should be used to normalize MDA concentrations [21,79]. Creatinine normalization is appropriate whenever spot urine samples are collected, while 24 h urine samples do not need adjustments [49]. Reporting efforts also include the descriptive statistics provided (GM and GSD): for this, we suggest reporting the median and the first and third quartile for a better interpretation of GSDs. In terms of analytical methods, we recommend using the DNPH derivatization agent over TBA and reporting both storage time and temperature.

## 5. Conclusions

Our systematic review and meta-analysis indicate a general population concentration range of 0.07–0.12 mg/g creatinine for GMs of urinary MDA in healthy adults. These GMs increase with the mean age of the study populations and cannot be used for comparison with individual results. There were several challenges encountered when analyzing the published data. The lack of homogeneity in data collection and storage conditions likely affected our meta-analysis. Consequently, the values determined in this study should be considered preliminary as they are based on moderate to low-quality studies. Further research efforts regarding the use of urinary MDA as a biomarker for oxidative stress need to address the following: standardize the reporting, understand the ideal urine collection time, elucidate optimal sample storage temperature, and the best derivatization agent as well as harmonize the chemical analytical methods.

## Figures and Tables

**Table 1:** Model-based estimates of geometric mean of urinary MDA concentrations (mg/g creatinine) by age group.

	GM	95% CI	Age Group	GM	95% CI
Overall	0.10	0.07–0.12	<30	0.05	0.03–0.10
			30–40	0.09	0.06–0.13
			40–50	0.13	0.08–0.17
			>50	0.12	0.09–0.18

**Table 2:** Results of the mixed-effect regression analyses according to geographical location, smoking status, and mean age of the population.

logGM	Coef.	Std. Err.	P > |z|	(95% Conf. Interval)	
**CatCountry**					
Latin America (n = 1)	−0.324	0.339	0.340	−0.988	0.341
North America (n = 9)	0.283	0.156	0.070	−0.024	0.590
China/Taiwan (n = 10)	0.490	0.164	0.003	0.169	0.812
Korea (n = 10)	0.421	0.199	0.034	0.032	0.811
Other Asia (n = 4)	0.384	0.257	0.134	−0.119	0.890
Europe (n = 13)	0	(base)			
**SmokCat**					
non-smok (n = 17)	0	(base)			
Mixed (n = 6)	0.008	0.084	0.922	−0.157	0.174
Smokers (n = 5)	0.102	0.061	0.096	−0.018	0.221
Not reported (n = 19)	0.389	0.141	0.006	0.113	0.665
**MeanAgeCat**					
<30 (n = 8)	0	(base)			
30–<40 (n = 11)	0.240	0.175	0.170	−0.103	0.584
40–<50 (n = 11)	0.369	0.181	0.042	0.014	0.723
>=50 (n = 17)	0.403	0.177	0.023	0.056	0.750
_cons	−1.75	0.181	0.000	−2.107	−1.40

## Data Availability

Not applicable.

## Supplementary Materials

The following supporting information can be downloaded at: https://www.mdpi.com/article/10.3390/toxics10040160/s1, Table S1: Quality assessment Criteria; Table S2: Quality appraisal.

## Abbreviations

MDA	Malondialdehyde
TBARS	Thiobarbituric acid reactive substances
ROS	Reactive oxygen species
GM	Geometric mean
GSD	Geometric standard deviation
BMI	Body mass index
SD	Standard deviation
SEM	Standard error of the mean
CV	Coefficient of variation
IQR	Interquartile range
