# Supplementary material for: Urinary Malondialdehyde (MDA) Concentrations in the General Population—A Systematic Literature Review and Meta-Analysis
Source: Toxics. 2022 Mar 29;10(4):160. doi: 10.3390/toxics10040160 (PMC9024833; doi:10.3390/toxics10040160)
Supplement: Supplementary file 1 [file toxics-10-00160-s001.zip › toxics-1624285 supp.pdf]

toxics

MDPI

Supplementary Materials: Urinary Malondialdehyde (MDA) Concentrations in the General Population—A Systematic Literature Review and Meta-Analysis

Antonio Toto, Pascal Wild, Mélanie Graille, Veronica Turcu, Camille Crézé, Maud Hemmendinger, Jean-Jacques Sauvain, Enrico Bergamaschi, Irina Guseva Canu and Nancy B. Hopf

Table S1. Quality assessment criteria.

Criteria for quality appraisal	Instructions for assessment	Number of points for Quality scoring		
		1 point	2 points	3 points
I. Study sample quality				
I.1. Representativeness of the study sample	The sample design and sample construction strategy should be clearly described in the article. If not, please consider the inclusion and exclusion criteria	Convenience sample	Consecutive sample	Probability sample (simple random sample, systematic sample, stratified random sample or cluster sample)
I.2. Between group comparability and potential for selection bias	To appraise this criteria, please consider the inclusion and exclusion criteria in each of the groups considered, depending on the study design (exposed/ unexposed or healthy/ ill) and the comparability of the groups finally selected in terms of all criteria except the exposure or the health outcome (statistical tests or sample descriptive statistics)	The cases and controls or exposed and unexposed participants are selected from the different settings and present different social, demographical, and biological characteristics (p-values < 0.05 or not reported, or not assessed)	An intermediate situation, where the groups are somehow comparable, because of the same criteria or the target/ accessible population used for the participant selection, and somehow comparable descriptive statistics between groups (no statistically significant difference for most of the characteristics between groups	The cases and controls or exposed and unexposed participants are selected from the same population, based on the same inclusion/ exclusion criteria and participation rate is similar in the both groups. Moreover, the statistical comparison of the groups did not reveal any statistically significant difference in terms of participant characteristics other than the exposure or the outcome, depending on the study design
I.3. Sample size and statistical power	To appraise this criteria, please consider the number of subjects in reference and in each group should be considered for the appraisal of this criteria	< 20	20–50	≥ 50
II. Study design quality				
II.1. Potential of confounding bias	To appraise this criteria, please consider how the authors address the	The reported results are significantly different with	The reported results are not significantly different with respect	The cases and controls were matched on these factors, or the results

	potential confound for at least three following factors: Age-, Sex-, Smoking status (1 = significantly different or no data collected; 2 = not statistically different; 3 = matched or reported by risk factor group	respect to the age, sex and smoking status or no data on them were collected	to the age, sex and smoking status	are reported by risk factor group (as result of the analyses stratified by age, sex and smoking status)
II.2.a Potential for misclassification bias on the exposure	To appraise this criteria, please consider how measurement of the exposure was done and which data were used for assessing cumulative exposure. If the study design is based on the healthy/ ill participants, please address the criteria II.2b	Assessed by researcher, without individual data, or with only qualitative individual data (questionnaires)	Assessed by the research team including hygienists air measurements and individual data on duration of employment	Assessed based on biological monitoring measurements and individual work history data
II.2.b Potential for misclassification bias on the outcome	To appraise this criteria, please consider how the diagnosis of disease was done	The cases were identified using existent medical records	The cases were examined by a study physician and confirmed as such	All study subjects were examined by the expert physician, and the diagnosis of each case was confirmed using the reference diagnostic procedure
III. Analytical/ technical quality	To appraise this criteria, please consider how thoroughly the laboratory analysis were conducted, in particular whether the author described the comparability of assessment methods if there is more than one group, the solvents used, the detector parameters (e.g., masse, UV-V), the internal or external calibration, and in case of internal calibration, the name/ RT of the compounds (except for immuno-essays), the LOD, LOQ, linearity, specificity, precision, accuracy, recovery rate, ,description of matrix effect, contamination, and criteria for acceptance of the method	Only LOD and LOQ were specified	Only 1/3 - small half of criteria were specified	More than half or 2/3 of the criteria were specified

III.1. Quality of laboratory analysis				
III.2 Measurements by mass spectrometry	Please assign the number of points accordingly to the specifications provided	Only succinct commentary of the method used was provided	The author followed and reporting the recommended guidelines used for their measurements	Full description of the validation of the method and guideline used, transitions, quantifications ions, confirmations ions, retention time of compounds, in case of internal calibration, the name/ RT of the compounds performed was reported
III.3. Measurements by ELISA or other techniques	Please assign the number of points accordingly to the specifications provided	Only succinct commentary of the method used was provided	The author set up their own technique and reported their validated analytical performance	The authors used a commercialized kit recognized as reference used
IV. Quality of the data processing, analysis and result reporting	To appraise this criteria, please consider how thoroughly the statistical methods were described, including the methods used to examine subgroups, confoundings, effect modifications and interactions, how missing data were addressed, and how the statistical significance was assessed. Please consider if the confidence intervals were given for the main results	Only succinct commentary of the method used was provided	Only some of criteria were specified and only p-values were reported for the significance of the results	More than 2/3 of the criteria were specified and confidence intervals were reported for the main results

Table S2. Quality appraisal.

**Articles**	**1**	**2**	**3**	**4**	**5**
**Quality Criteria/ Studies**	**Adetona A.M.**	**Adetona O.**	**Argawal R.**	Argawal R.-Shawn D.	Akbari M.
**I. Study sample quality**					
I.1. Representativeness of the study sample	2	2	2	2	2
I.2. Between group comparability and potential for selection bias	2	2	2	2	2
I.3. Sample size and statistical power	1	1	1	1	2
**II. Study design quality**					
II.1. Potential of confounding bias	3	3	2	2	3
II.2.a Potential for misclassification bias on the exposure	2	1	1	1	1
II.2.b Potential for misclassification bias on the outcome	3	3	2	2	2
**III. Analytical/ technical quality**					
III.1. Quality of laboratory analysis	0	0	0	0	0
III.2 Measurements by mass spectrometry	2	2	2	2	1
III.3. Measurements by ELISA or other techniques	0	0	0	0	0

**IV. Quality of the data processing, analysis and result reporting**	3	2	2	3	3
**Final score** *(sum of the points assigned to each of above-cited criteria, the highest score is 27 (3 points assigned to each of 9 criteria)*	18	16	14	15	16
**Quality level (< or = 13 = Low; 14 to 19 = Moderate; > 20 = High)**	Moderate	Moderate	Moderate	Moderate	Moderate

*studies included in the meta-analysis.

**6**	**7**	**8**	**9**	**10**	**11**	**12**	**13**	**14**	**15**
Aksu N.	Allen R. W.	Al-Saleh I.	Anderson G. W.	Ates I.	Bellisario V.	Bin P.	Boonla C.	Boudouris G.	Boyle S.P.
2	2	2	2	2	2	2	2	1	2
2	2	2	2	1	2	2	2	1	2
3	2	3	1	3	3	3	3	1	1
2	2	2	2	1	2	2	2	2	2
2	1	1	2	1	1	1	1	2	1
2	1	2	2	1	2	1	1	3	1
0	0	2	0	0	0	0	0	0	0
0	0	2	2	1	0	0	3	1	1
1	1	0	0	0	1	3	0	0	0
2	3	2	2	2	2	2	2	1	3
16	14	18	15	12	15	16	16	12	13
Moderate	Moderate	Moderate	Moderate	Low	Moderate	Moderate	Moderate	Low	Low
